# Sepsis by *Pasteurella multocida* in an Elderly Immunocompetent Patient after a Cat Bite

**DOI:** 10.1155/2017/2527980

**Published:** 2017-11-26

**Authors:** Lara Caserza, Gabriella Piatti, Aldo Bonaventura, Luca Liberale, Federico Carbone, Franco Dallegri, Luciano Ottonello, Giulia Gustinetti, Valerio Del Bono, Fabrizio Montecucco

**Affiliations:** ^1^First Clinic of Internal Medicine, Department of Internal Medicine, University of Genoa, 6 Viale Benedetto XV, 16132 Genoa, Italy; ^2^Ospedale Policlinico San Martino, 10 Largo Benzi, 16132 Genoa, Italy; ^3^Department of Surgical and Diagnostic Sciences, Section of Microbiology, University of Genoa, 8 Largo Benzi, 16132 Genoa, Italy; ^4^Center for Molecular Cardiology, University of Zürich, 12 Wagistrasse, Schlieren, CH-8952 Zürich, Switzerland; ^5^Clinica Malattie Infettive, DIPMI, DISSAL, University of Genoa, 10 Largo Benzi, 16132 Genoa, Italy; ^6^Centre of Excellence for Biomedical Research (CEBR), University of Genoa, 9 Viale Benedetto XV, 16132 Genoa, Italy

## Abstract

*Pasteurella multocida* colonizes animal scratches and bites. This bacterium was described to cause sepsis or endocarditis mainly in immunocompromised patients. We report the case of a 92-year-old woman presenting at the Emergency Department with coma and fever a week after the bite of her cat. The cat bite was misdiagnosed at admission partly due to an underestimation of this event by the patient's relatives. An inflamed area localized at perimalleolar skin of the right leg was detected. Laboratory biomarkers of inflammation were elevated. The cerebral computed tomography (CT) scan with angiographic sequences showed a complete occlusion of right intracranial vertebral artery. Total body CT scan and abdominal echocardiography were negative for foci of infection. Three consecutive blood cultures were positive for *Pasteurella multocida*. A diagnosis of sepsis by *Pasteurella multocida* was made, and the patient recovered after a specific antimicrobial treatment. In order to confirm the animal transmission, the cat saliva was cultured and found positive for *Pasteurella multocida* with a similar antibiotic sensitivity to that isolated from the patient. In conclusion, the case of a patient with coma and fever after a cat bite was presented. The transmission of pathogens from pets has to be carefully considered as an important route of infection in immunocompetent patients.

## 1. Introduction

*Pasteurella multocida* is a Gram-negative coccobacillus frequently isolated in the upper respiratory tract and gastrointestinal microbiota of many animals. Specifically, cats showed the highest carriage rate among pets, ranging from 70 to 90%. Animal scratches and bites are the most common sources of human *Pasteurella* infections and expression of the disease may vary from mild local symptoms to life-threatening conditions, such as septic shock. We present a case of sepsis and coma in an elderly immunocompetent woman due to *Pasteurella multocida* after a cat bite.

## 2. Case Report

**Figure 1:**
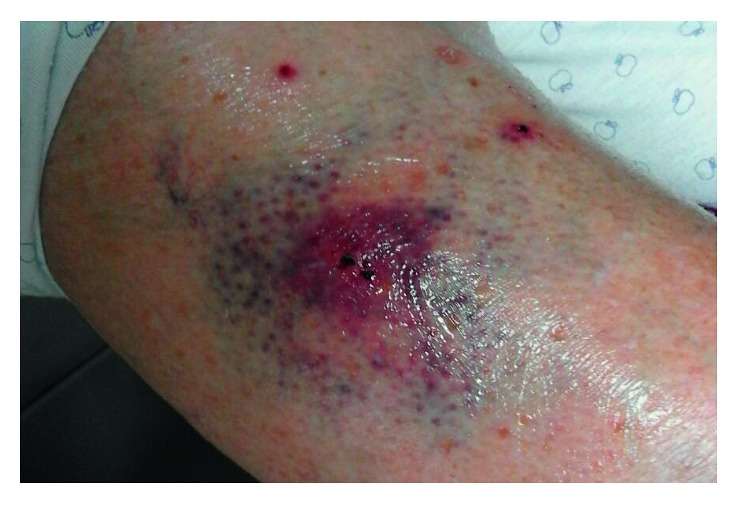
The cat bite on the right leg of the patient.

**Figure 2:**
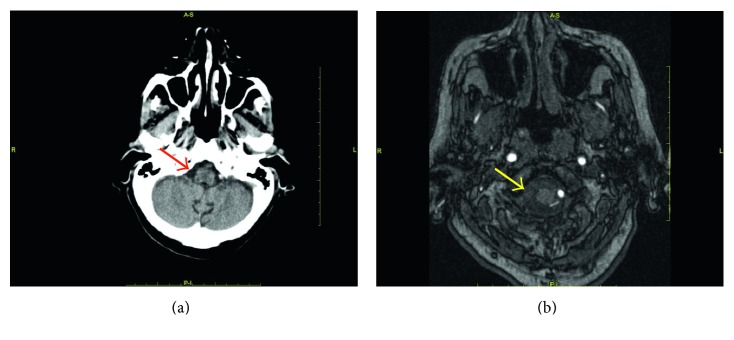
Brain CT scan (a) and MRI (b) showing the complete occlusion (arrows) of the right intracranial vertebral artery.

A 92-year-old woman was accompanied to the Emergency Department in a coma status (Glasgow Coma Scale (GCS) of 7) with feces loss and fever (39.5°C). Blood pressure was 180/110 mmHg, the respiratory rate was 25 breaths/minute, and arterial blood oxygen saturation was 92% while breathing on room air. Furthermore, the physical examination revealed a systolic mitralic murmur and an inflamed area on perimalleolar skin on the right leg (Figure 1). The personal medical history included hypertension, mild cognitive impairment related to vascular encephalopathy, chronic atrial fibrillation, chronic heart failure, and bleeding complications after falls during anticoagulant therapy. The patient did not take any immunosuppressive or anti-inflammatory drug at home. At admission, the electrocardiogram (ECG) confirmed atrial fibrillation with normal heart rate. Laboratory blood tests demonstrated a white blood cell count (WBC) of 13,160/mm^3^, haemoglobin of 159 g/L, lactic acid of 2.5 mmol/L, creatinine of 0.7 mg/dL, troponin I of 0.238 μg/L, C-reactive protein (CRP) of 6.2 mg/L, and lactic dehydrogenase of 252 U/L. The brain computed tomography (CT) scan with angiographic sequences demonstrated the complete occlusion of the right intracranial vertebral artery, diffused signs of leukoencephalopathy, and no haemorrhages (Figure 2). The brain magnetic resonance imaging (MRI) excluded a recent cerebral ischemia but confirmed the occlusion of the right intracranial vertebral artery (Figure 2). Total body CT scan was not able to detect any macroscopic foci of infection or cancer. The Doppler ultrasound examination of leg arteries and veins as well as carotid arteries did not detect any thrombosis and/or stenosis. Blood cultures were performed and, while waiting for the isolation time, an empirical antimicrobial treatment with piperacillin/tazobactam was started together with supportive therapies. No significant clinical improvement was observed, and inflammatory biomarkers, such as procalcitonin (with a peak of 4.0 µg/L) and CRP (with a peak of 200 mg/L), increased. On the other hand, troponin I blood concentration rapidly reached the normal values.

A targeted antimicrobial therapy with ampicillin-sulbactam was immediately started, in association with gentamicin in order to obtain a synergistic antimicrobial activity in consideration of the possible diagnosis of endocarditis. After one day of such a therapy, the patient's consciousness restored and the fever stopped. On day four after the admission, three blood cultures resulted positive for Gram-negative coccobacilli. In the Microbiology Laboratory, blood samples were collected and inoculated in BD BACTEC™ Plus Aerobic/F and Anaerobic/F culture vials and incubated in the automated system BD BACTEC. Positive aerobic specimens were seeded on blood and chocolate agar plates, giving round, grey, nonhaemolytic, nonmucoid pure colonies after 24 hours. Bacterial stain was identified as *Pasteurella multocida* with the automated biochemical testing Vitek 2 (BioMeriéux Italia S.p.A., Grassina, Italy), which also performed antibiotic sensitivity, indicated in Table 1. Identification was confirmed with the matrix-assisted laser desorption ionization time-of-flight mass spectrometry (MALDI-TOF) VITEK MS (BioMeriéux Italia S.p.A.). The transthoracic echocardiogram on day 10 did not show any endocarditic lesions, and the patient completely recovered. In the meanwhile, the patient could refer about the skin lesion due to her cat bite, occurring one week before her hospital admission. Since the cat was still living at the patient's home, a sample of its saliva was analyzed.

In particular, a cat pharyngeal swab was performed and seeded on blood and chocolate agar plates and grew at 37°C in aerobic condition. After 24 hours, mixed bacterial colonies grew, from which an isolation on blood agar was performed to grow in anaerobic condition. The anaerobic growth gave the sole colonies the same look as the ones isolated from patient blood culture. Colonies from cat were identified as *Pasteurella multocida* through the same technologies, that is, the automated biochemical testing and the mass spectrometry. The antibiotic sensitivity of cat strain was analyzed with the Vitek system as well. The phenotype of sensitivity of the patient strain and cat strain was the same, and the related MIC values were similar (Table 1). In particular, cefotaxime MIC value of patient isolates was < 1 mg/L, while that of cat isolates was 1 mg/L. Such a quantitative diversity corresponds to the variability achievable with a strain alone (Table 1).

The patient was discharged after 15 days of antimicrobial therapy (ampicillin/sulbactam and gentamicin). Additional blood cultures during antibiotic treatment were negative, and procalcitonin and CRP levels were reduced to 0.09 µg/L and 33.6 mg/L, respectively. Additional 15-day treatment with amoxicillin and clavulanic acid was recommended at home. During hospitalization, the patient underwent anticoagulation therapy for elevated ischemic stroke risk (CHA₂DS₂-VASc Score: 7 points) since the beginning of deambulation. Then, due to the HAS-BLED Score 3 points, the high risk of falls and previous fall-related major bleeding complications during anticoagulation therapy, the patient was discharged without an anticoagulant therapy. After one-year follow-up, the patient regained her prehospitalization life without any additional neurological impairment. However, she preferred to avoid living with a pet. The patient formally approved the publication of this anonymized case report and signed the informed consent.

## 3. Discussion

Skin infections due to cat bites are commonly due to *Pasteurella*. Rarely, the local disease can lead to sepsis or septic shock [1–5]. In general, people affected by these severe forms are aged over 65 years or have comorbidities (such as diabetes and immunodepression) [3, 6, 7]. After a cat bite, a proper cleaning of the wound and the initiation of a broad spectrum antibiotic therapy are recommended. Since animal bites potentially deliver a polymicrobial infection [8–10], a combination of amoxicillin and the β-lactamase inhibitor (clavulanic acid) or doxycycline plus metronidazole (for patients with penicillin allergies), or clindamycin plus a fluoroquinolone (i.e., ciprofloxacin), should be recommended [10]. Rarely, penicillin-resistant *Pasteurella* was described in human infections [11]. Since this kind of infection can be treated with common antibiotics at home, many people consider cat bites and scratches not dangerous and do not advise family physician or do not refer to this event when admitted to hospital.

In the abovementioned case report, the patient did not suffer from diabetes or any other known immunodeficiency. At the complete interview, she reported to be bitten twice from her cat in the previous month: the first time presenting an acute local infection that spontaneously recovered, and the second time with a septic fever needing hospitalization. We might also speculate on the vertebral artery thrombosis diagnosed by brain CT scan and MRI, although clinically silent. This finding could be attributed either to a septic embolus or to a cardio embolization related to chronic atrial fibrillation. Since the patient recovered, she preferred not to perform any additional neuroimaging during follow-up. This point remains to be clarified. Interestingly, our patient did not respond to piperacillin-tazobactam treatment, even though piperacillin shows a good in vitro activity against *Pasteurella* [12]. We do not have any clear explanation for this finding. Possibly, the dosage used in our case (2.25 g three times per day calculated on the creatinine clearance) did not allow to reach effectively therapeutic plasma levels. Sepsis by *P. multocida* is rare but not an extraordinary event. Thus, our interest just emerged after the awareness of the possible link between sepsis and the wound due to a cat bite. Having not collected the patient strain, we could not perform comparative sequence analysis to definitively confirm that the *P. multocida* source was from the cat. We used the similarity between the antibacterial sensitivity phenotype of the strains, from the patient and cat, to presume that the identity of microorganisms was the same. This trait, along with the lacking of strains serotyping, is a limit of our report. Anyway, the temporal concordance of the events and the diagnostic elements in our possession allow us to think that the correlation we posed is not risky and worthy of being notified. From the microbiological point of view, it is interesting to note that we achieved isolation of *Pasteurella*, among cat pharyngeal microbiota, only by incubating blood and chocolate agar plates in anaerobic conditions, without the employment of selective media. It is likely that, being a facultative anaerobe organism, *P. multocida* has benefited from conditions unfavorable for other bacteria present in the cat oral mucosa. In conclusion, the cat bites and scratches can drive many pathogenic bacteria, such as *Pasteurella multocida*, and cause deep wounds, especially on the legs and arms. Usually, these lesions are treated with a local approach and are often underestimated by people. Only in few circumstances, patients require a medical examination and begin a proper antibiotic treatment. In elderly patients, especially when affected by other comorbidities, cat bites can lead to local complications or even to a *Pasteurella multocida* sepsis, the latter being an unusual, albeit severe condition. A wider awareness of this problem may be useful in order to prevent life-threatening conditions after pet bites [13], especially in elderly people. The importance of a prompt antibiotic administration should also be underlined. In case of fever after cat bite or scratch and suspected poor compliance to antibiotic therapy, hospitalization may be required.

## Figures and Tables

**Table 1:** Antibiotic sensitivity phenotype of *Pasteurella multocida* strains from the patient and cat, in mg/L of minimal inhibitory concentration (MIC).

Antimicrobial drug	Patient	Phenotype	Cat	Phenotype
Amoxicillin/clavulanic acid	≤2	S	≤2	S
Piperacillin/tazobactam	≤4	S	≤4	S
Cefotaxime	≤1	S	1	S
Ceftazidime	2	S	2	S
Ertapenem	≤0.5	S	≤0.5	S
Imipenem	≤0.25	S	≤0.25	S
Meropenem	≤0.25	S	≤0.25	S
Amikacin	16	I	16	I
Gentamicin	4	I	4	I
Ciprofloxacin	≤0.25	S	≤0.25	S
Tigecycline	≤0.5	S	≤0.5	S
